# Knowledge and Attitude of Newly Diagnosed Breast Cancer Patients and Their Accompanying Attendants About Multimodality Treatment for Breast Cancer

**DOI:** 10.7759/cureus.7915

**Published:** 2020-05-01

**Authors:** Nahan Siddique Ruby, Chellappa Vijayakumar, Sudharsanan Sundaramurthi, Sathasivam Sureshkumar, Uday Kumbhar, Gopal Balasubramanian

**Affiliations:** 1 Surgery, Jawaharlal Institute of Postgraduate Medical Education and Research (JIPMER), Puducherry, IND

**Keywords:** awareness, knowledge, attitude, multi-modality treatment, breast cancer

## Abstract

Introduction

This study was done to estimate the level of knowledge and attitude about the multimodality treatment (MMT) of breast cancer among the newly diagnosed breast cancer patients and accompanying attendants. Apart from the lack of knowledge, it is equally important to consider their accompanying attendant's knowledge, which changes the patient's attitude.

Methodology

This was a cross-sectional analytic study, including all newly diagnosed breast cancer patients of age above 18 years. The initial questionnaire one (Q1) was about their overall knowledge of carcinoma breast treatment options. Subsequent questionnaire two (Q2) were asked about MMT for breast cancer at eliciting their attitudes about MMT. After explaining about MMT for breast cancer, they were asked to indicate if they had a positive/negative attitude about MMT by questionnaire three (Q3).

Results

A total of 84 patients was included in the study. The results indicate a significant association between the relation of the attendant to the patient and their level of knowledge of MMT (p<0.001). Approximately 62% of study patients preferred a passive role in making their treatment decisions of MMT, with nearly 26.2% preferring their treating doctor to make all decisions while 36.7% preferred decisions by accompanying attendants. None of the patients had a negative attitude about MMT.

Conclusion

The treating surgeon should analyse the patient's knowledge of MMT and their attitude toward involving their accompanying attendants in making MMT decisions. It is necessary to administer adequate knowledge regarding MMT and discuss the various treatment options for breast cancer with the ailing patient, along with the accompanying attendants.

## Introduction

Providing adequate knowledge to patients about breast cancer helps them cope with cancer treatment [1]. The accompanying attendant role is important among the other key participants for a patient with breast cancer [2]. Knowledge and attitude towards multimodality treatment (MMT) also differ among different patient populations [3-4]. Family participation in decision making and communication is essential for cancer patients especially in developing countries like India [5]. The solution to active participation in breast cancer management lies in providing adequate knowledge about MMT of breast cancer. Apart from a lack of knowledge, it is equally important to consider their accompanying attendant's knowledge, which changes patient's attitudes [6]. This study was done to estimate the level of knowledge and attitude about MMT for breast cancer among the newly diagnosed breast cancer patients and their accompanying attendants. This article was first presented for an undergraduate research award (Golden Jubilee Short-Term Research Award to undergraduate students-GJSTRAUS) at the Fifth Annual Research Day 2020, Jawaharlal Institute of Postgraduate Medical Education and Research (JIPMER), Pondicherry, India, January 10, 2020 (https://www.jipmer.edu.in/sites/default/files/Souvenir-5th%20JIPMER%20Research%20Day%20Jan%202020.pdf).

## Materials and methods

This cross-sectional analytical study was done at a tertiary care centre in South India for a period of six months (March 2019 to August 2019). Approval was taken from the institute's ethics committee (Human Studies). Study patients were all newly diagnosed breast cancer patients of age above 18 years attending the outpatient department (OPD). The study excluded breast cancer patients presenting to the emergency department with complications, terminally ill breast cancer patients, and patients partially treated elsewhere. Patients and their accompanying attendants were interviewed with a standardized questionnaire after obtaining informed consent. The questionnaire used was validated in the Indian set up [7].

The patients and accompanying attendants were asked to select one of the responses from A to F statements as mentioned in this study results. The first two statements (A and B) and the third statement (C) were considered an active role and a collaborative role in the attitude of their MMT, respectively. Similarly, the next two statements (D and E) and the final statement (F) were considered a passive role and negative role in the attitude of their MMT respectively. Data regarding the patient's and the accompanying attendant's knowledge of MMT, educational status, income, occupation, marital status, and residence were obtained by interviewing them. Data regarding diagnosis (stage of breast cancer), treatment (MMT) status, time since diagnosis, and comorbidities were obtained from case records.

Statistical analysis

Categorical variables were expressed as proportions in the analysis. The association with other factors was analysed using the chi-square test. Multivariate logistic regression could not be done because of the small sample size. A p-value of <0.05 was considered statistically significant. The analysis was done by using statistical software IBM SPSS version 21.0 (IBM Corp., Armonk, NY).

## Results

A total of 42 patients gave consent to participate in this study. The mean age of the study patients was 45.7 years. The majority of breast cancer patients (n=26: 61.9%) were from urban areas. Of all the study patients, only two (4.76%) were graduates. All the patients were married. Accompanying attendants of all the 42 patients gave consent to participate in this study. The mean age of the accompanying attendants was 37.5 years. The majority of the accompanying attendants (n=31; 73.8%) were from urban areas. Of all the accompanying attendants, 10 (23.8%) were graduates. The majority of the accompanying attendants (n=32; 76.19%) were directly related to the patient, either husband or son/daughter.

Among the study patients, 29 (69.05%) had partial knowledge of the treatment of breast cancer and the specific meaning of MMT but they wanted further details and individual aspects of MMT. The proportion of patients who partially knew, but would like to know, about the week-by-week progress of MMT, chances of a cure if not treated by MMT, components of MMT and side effects of MMT were 64.3%, 76.2%, 66.7%, and 61.9%, respectively. The proportion of patients who want to know exactly how MMT works to treat breast cancer was 76.2% in this study. Among the accompanying attendants, 30 (71.5%) and 33 (78.6) partially knew about the treatment of breast cancer and the specific meaning of MMT, but they wanted further details and individual aspects of MMT. The proportion of accompanying attendants who partially knew but would like to know about the week-by-week progress of MMT, chances of a cure if not treated by MMT, components of MMT, and side effects of MMT were 81%, 83.3%, 73.8%, and 73.8%, respectively. The proportion of accompanying attendants who want to know exactly know how MMT works to treat breast cancer was 78.8% in this study.

On studying the knowledge level, an average level (7-9) of knowledge of MMT was found in 23 (54.8%) patients and 29 (69%) accompanying attendants. A very good level (>13) of knowledge was studied in three (7.1%) patients and in one (2.4%) attendant (Table 1).

**Table 1:** Level of knowledge of MMT of the patients and accompanying attendants MMT: multimodality treatment

Level of Knowledge	Patients [N (%)]	Accompanying attendants [N (%)]
Very good ( >13)	3 (7.1)	1 (2.4)
Good (10 -12)	3 (7.1)	3 (7.1)
Average (7-9)	23 (54.8)	29 (69)
Poor (4-6)	5 (11.9)	3 (7.1)
Very poor (< 3)	8 (19)	6 (14.3)

Regarding participation in treatment after knowing about MMT by making individual/combined decisions, 26 patients (61.9%) preferred a passive role, nine patients (21.4%) preferred an active role, and seven (16.7%) preferred a collaborative role. None of the patients had a negative attitude towards MMT (Table 2).

**Table 2:** Attitude of patients to participate in treatment after knowing about MMT A, B, C, D, E, F: different patient statements; MMT: multimodality treatment

Statement	N (%)	Preference for MMT decision, [N (%)]
A. I prefer to make the final selection about which treatment I will receive	6 (14.3)	Active role, 9 (21.4)
B. I prefer to make the final selection of my treatment after seriously considering my doctor’s opinion	3 (7.1)	
C. I prefer that my doctor and I share responsibility for deciding which treatment is best for me	7 (16.7)	Collaborative role, 7 (16.7)
D. I prefer that my doctor make the final decision about which treatment, but seriously considers my opinion	11 (26.2)	Passive role, 26 (61.9)
E. I prefer to leave all decisions regarding treatment to my accompanying attendants	15 (35.7)	
F. I don’t want to continue any type of treatment in future	0	Negative attitude, 0

Regarding participation in treatment after knowing about MMT by making individual/combined decisions, 23 accompanying attendants (54.8%) preferred an active role, 13 accompanying attendants (31%) preferred a collaborative role, and six (14.3%) preferred a passive role. None of the accompanying attendants had a negative attitude towards MMT (Table 3).

**Table 3:** Attitude of accompanying attendants to participate in treatment after knowing about MMT A, B, C, D, E, F: different accompanying attendant statements; MMT: multimodality treatment

Statement	N (%)	Preference for MMT decision, [N (%)]
A. I prefer to make the final selection about which treatment she will receive	11 (28.6)	Active role, 23 (54.8)
B. I prefer to make the final selection of her treatment after seriously considering my some more relative’s opinion	12(26.2)	
C. I prefer that another relatives and I share responsibility for deciding which treatment is best for her	13 (31)	Collaborative role, 13 (31)
D. I prefer that another relative’s make the final decision about which treatment, but seriously considers my opinion	1 (2.4)	Passive role, 6 (14.3)
E. I prefer to leave all decisions regarding treatment to another relative	5 (11.9)	
F. I don’t want to continue any type of treatment in future	0 (0)	Negative attitude, 0(0)

There was no significant association between secondary factors like age, residence, education, income, marital status, performance status, time since diagnosis, and treatment plan with the level of knowledge about MMT. There was a significant association between close relatives like husband/son/daughter and the average level of knowledge (N=23; 71.87%; p=0.001). Other than this, there was no significant association between the secondary factors of attendants like age, residence, education, income, marital status, performance status, time since diagnosis, treatment plan, and level of knowledge about MMT.

The patient attitude scale also did not show any significant association with demographic variables, stage of the disease, treatment plan, performance status and time since diagnosis. Patients who were awaiting neo-adjuvant treatment and palliative care preferred a collaborative decision with their accompanying attendants when compared to those who were to undergo upfront surgery and those who were in the early breast cancer stage (Table 4).

**Table 4:** Association of the demographic profile and attitude of the patients with MMT decisions A, B, C, D, E, F: different patient statements; MMT: multimodality treatment

Parameter [N(%)]		A & B (Active role)	C (Shared role)	D & E (Passive role)	F (Negative Role)	p-value
Age	<40	3 (42.8)	2 (28.6)	2 (28.6)	0	0.134
	>40	6 (17.1)	5 (14.3)	24 (68.6)	0	
Residence	Urban	3 (18.7)	4 (25)	9 (56.2)	0	0.523
	Rural	6 (23)	3 (11.5)	17 (65.4)	0	
Education	Primary School	0	2 (33.3)	4 (66.7)	0	0.266
	High School	5 (27.8)	3 (16.7)	10 (55.5)	0	
	College	1 (50)	1 (50)	0	0	
	None	3 (18.7)	1 (6.25)	12 (75)	0	
Income	<2000	4 (23.5)	0 (0)	13 (76.5)	0	0.055
	>2000	5 (20)	7 (28)	13 (25)	0	
Marital status	Married	9 (21.4)	7(16.7)	26 (61.9)	0	-
	Unmarried	0	0	0	0	

The accompanying attendant’s scale also did not show any significant association with demographic variables, stage of the disease, income, relation with patient, and treatment plan. Attendants of patients who were awaiting neo-adjuvant treatment and palliative care preferred a collaborative decision with their treating surgeon when compared to those who were undergoing upfront surgery and those who were in the early breast cancer stage (Table 5).

**Table 5:** Association of the demographic profile and attitude of the accompanying attendant with MMT decisions A, B, C, D, E, F: different accompanying attendant statements; MMT: multimodality treatment

Parameter [N (%)]		A & B (Active role)	C (Shared role)	D & E (Passive role)	F (Negative Role)	p-value
Age	<40	15 (62.5)	6 (25)	3 (12.5)	0	0.502
	>40	8 (44.4)	7 (38.8)	3 (16.7)	0	
Residence	Urban	6 (54.5)	5 (45.4)	0	0	0.208
	Rural	17 (68)	8 (32)	0	0	
Education	Primary School	2 (66.7)	1 (33.3)	0	0	0.604
	High School	15 (55.6)	9 (33.3)	3 (11.1)	0	
	College	6 (60)	2 (20)	2 (20)	0	
	None	0	1 (50)	1 (50)	0	
Relationship	Husband	19 (59.4)	8 (25)	5 (15.6)	0	0.653
	Mother	1 (33.3)	2 (66.7)	0	0	
	Sister	2 (40)	2 (40)	1 (20)	0	
	Friend	0	1 (100)	0	0	
	Relatives	1 (100)	0	0	0	

## Discussion

The patient's demographic parameters did not affect the level of knowledge about MMT. Similarly, the accompanying attendant’s demographic parameters also did not affect their MMT knowledge. Many studies concluded that most of the young, educated patients wanted to know about MMT with a positive attitude towards their treatment [8-9]. Although the majority of this study's patients and the accompanying attendants had a lower level of education, they wanted to know about MMT.

In this study, approximately 16.6% preferred a collaborative role in their MMT. This is in contrast to the western studies where more patients preferred a collaborative role in their treatment [9-10]. Singh et al. in their study found that more than 50% of patients preferred a collaborative role with their treating surgeons [4]. Similarly, Schaede et al. and Breura et al. in their studies showed that 63% of patients preferred a collaborative role while 20% preferred an active role and only 17% preferred a passive role in their treatment decisions [10-11]. In this study, similar results were seen with regards to the preference for the active role while there was an abrupt contrast in the number of patients preferring a collaborative or passive role. The majority of Indian patients prefer a passive role in their treatment due to a lower level of education and their preference to give importance to their health than their personal appearance [7,11]. Gilani et al. and Kraetschmer et al. in their studies found that patients and accompanying attendants prefer an active role when the trust levels are less with the treating surgeons [12-13]. Sankar et al. found that Indian patients and their accompanying attendants are used to a paternalistic approach when the trust levels are higher with their treating surgeon [7]. This may be the reason why the majority of this study's patients played a passive role in MMT.

The stage of breast cancer did not have a significant role in the patient's attitude. Similarly, the attitude of accompanying attendants did not have a significant association with baseline demographic factors. A collaborative role with the accompanying attendants has been shown to be associated with improved outcomes in breast cancer patients. Sankar et al. found that involving accompanying attendants increased patient satisfaction in their treatment pathway [7]. Few studies found that an active role controlled by family members was more common among Asians [7,13]. It is often seen from one responsible accompanying attendant who takes most of the health-related decisions in their family [7]. This may be the reason why a majority of this study patients preferred a collaborative role with their accompanying attendants. Many studies have found that Indian patients have the tendency to change their attitudes frequently [7,14]. Providing appropriate knowledge about MMT to accompanying attendants can facilitate their active participation. Hence, the accompanying attendant's involvement has been shown to be associated with improved outcomes in breast cancer patients. The strength of this study was that there was no data available about the attitude of breast cancer patient’s accompanying attendants towards MMT.

Limitations

This study was conducted in a single tertiary care institute in South India with a small sample size. Since most of the study patients were from poor socio-economic backgrounds, study results may not be represented by the other parts of India. Further prospective analytic studies with a large sample size are required to assess the knowledge and attitude among patients and accompanying attendants representing all socioeconomic levels.

## Conclusions

The majority of patients and their accompanying attendants want to know more about the MMT of breast cancer. Although patients have a desire to know more about MMT, their attitude towards MMT for breast cancer was more passive. The majority of patients with a passive role in treatment wanted their accompanying attendants to lead the active role in their treatment. Hence, providing appropriate knowledge about MMT to accompanying attendants can facilitate their better involvement, which in a country like India, with high family values, can influence the outcome of breast cancer patients.
